# Isolation and Molecular Characterization of Novel Chlorpyrifos and 3,5,6-trichloro-2-pyridinol-degrading Bacteria from Sugarcane Farm Soils

**DOI:** 10.3389/fmicb.2017.00518

**Published:** 2017-04-04

**Authors:** Smriti Rayu, Uffe N. Nielsen, Loïc Nazaries, Brajesh K. Singh

**Affiliations:** ^1^Hawkesbury Institute for the Environment, Western Sydney University, PenrithNSW, Australia; ^2^Global Centre for Land-based Innovation, Western Sydney University, PenrithNSW, Australia

**Keywords:** microbial bioremediation, chlorpyrifos, TCP, pesticides, degradation

## Abstract

Chlorpyrifos (CP) is one of the most widely used organophosphate pesticides in agriculture worldwide, but its extensive use has led to the contamination of various soil and water systems. Microbial bioremediation is considered to be one of the most viable options for the removal of CP from the environment; however, little is known about the soil bacterial diversity that degrade CP. Sequential soil and liquid culture enrichments enabled the isolation of bacterial CP degraders with sequence homologies to *Xanthomonas* sp., *Pseudomonas* sp., and *Rhizobium* sp. The efficacy of the three isolated strains: *Xanthomonas* sp. 4R3-M1, *Pseudomonas* sp. 4H1-M3, and *Rhizobium* sp. 4H1-M1 was further investigated for biodegradation of CP and its primary metabolic product, 3,5,6-trichloro-2-pyridinol (TCP). The results indicate that all three bacterial strains almost completely metabolized CP (10 mg/L) and TCP, occurring as a metabolic degradation product, in mineral salt media as a sole source of carbon and nitrogen. The isolated bacterial strains *Xanthomonas* sp. 4R3-M1 and *Pseudomonas* sp. 4H1-M3 could also degrade TCP (10 mg/L) as a sole carbon and nitrogen source, when provided externally. Thus, these bacterial strains may be effective in practical application of bioremediation of both CP and TCP.

## Introduction

Chlorpyrifos (CP) [*O,O*-diethyl *O*-(3,5,6-trichloro-2-pyridyl) phosphorothioate)], an organophosphate (OP) pesticide is one of the most widely used pesticides in agriculture against a broad spectrum of insect pests of economically important crops (Kale et al., 1999; Mallick et al., 1999; Fang et al., 2006; Singh and Walker, 2006). CP is a highly effective pesticide because it irreversibly inhibits acetylcholinesterase (AChE) enzymes leading to excess acetylcholine accumulation at nerve terminals resulting in agitation, hypersalivation, convulsion, and ultimately death of insects and mammals (King and Aaron, 2015). However, continuous and excessive use in the agricultural sector has resulted in contamination of soil, air, and ground- and surface-water (Sapozhnikova et al., 2004; Yang et al., 2005; Yu et al., 2006). The half-life of CP generally ranges between 10 and 120 days in soil but can be up to 1 year depending on abiotic factors such as temperature, moisture, pH, etc. (Howard, 1991; Singh and Walker, 2006). There is also a growing concern of widespread contamination of the environment leading to potential risks to non-target organism because of its entry in the food chain (Aysal et al., 2004; Chandra et al., 2010; Muhammad et al., 2010) and undesirable health issues to humans that include developmental toxicity, liver damage, reproductive defects, endocrine disruptions, nervous system disorders, and immune system abnormalities (Furlong et al., 2006; Rauh et al., 2011; Terry, 2012; Alavanja et al., 2013). The acute toxicity of CP has also been reported in some animals including aquatic invertebrates and fishes, arthropods, and soil microorganisms (Michereff-Filho et al., 2004; Palma et al., 2008; Tuzmen et al., 2008; Dutta et al., 2010).

In the environment, CP is degraded to 3,5,6-trichloro-2-pyridinol (TCP), which is the primary and major degradation product (Singh et al., 2003b, 2006; Yang et al., 2005; Kim and Ahn, 2009; Li et al., 2010). TCP is classified as a persistent metabolite by the US Environmental Protection Agency (US EPA) with a half-life ranging from 65 to 360 days in soil (Armbrust, 2001). The presence of three chloride atoms on the *N*-aromatic ring contributes to increased resistance of this metabolite to microbial degradation (Singh et al., 2003a; Chishti and Arshad, 2013; Jabeen et al., 2015). The released chlorine atoms from TCP have antimicrobial activity that prevents the proliferation of CP-degrading bacteria (Racke, 1993). Owing to TCPs’ greater persistence and water solubility compared to its parent compound, it leaches into the ground and surface water bodies causing widespread contamination of soils and aquatic environments, posing significant ecotoxicological risks (Feng et al., 1997; Xu et al., 2008; Watts, 2012). The Australian Pesticides and Veterinary Medicines Authority (APVMA) have identified CP and its metabolites to be assessed for spray drift risks due to human health and environmental concerns (Immig, 2010). Other sources of contamination are industrial effluents, disposal by consumers, leakages, and accidental spills that require large-scale decontamination. Therefore, developing technologies to remove CP and TCP from contaminated areas or disposal of manufacturing wastes are needed in order to minimize their adverse impact on human health, non-target organisms, and the environment.

Among the proposed remediation techniques of contaminated ecosystems, bioremediation is considered one of the most promising approaches. It is a relatively low cost, easy to use and environmentally friendly technique (Rayu et al., 2012). The use of microorganisms having the right metabolic pathways is one of the most viable options for the remediation of CP and TCP in soil and water (Li et al., 2010; Thengodkar and Sivakami, 2010; Singh et al., 2011). Previously, CP was reported to be resistant to degradation (Racke et al., 1990; Mallick et al., 1999), but later studies identified bacteria from the genera *Enterobacter* (Singh et al., 2004), *Pseudomonas* (Lakshmi et al., 2009; Farhan et al., 2012; Chawla et al., 2013), *Bacillus* (Liu et al., 2012; El-Helow et al., 2013), and *Klebsiella* (Ghanem et al., 2007) that were able to degrade CP efficiently. In other recent studies, it was further determined that some of CP-degrading bacterial strains from the genera *Bacillus* (Anwar et al., 2009), *Alcaligenes* (Yang et al., 2005), *Paracoccus* (Xu et al., 2008), *Gordonia* (Abraham et al., 2013), *Sphingobacterium* (Abraham and Silambarasan, 2013), and *Mesorhizobium* (Jabeen et al., 2015) could utilize CP as a sole source of carbon (C) and also degrade TCP. The first ever bacterium (*Pseudomonas* sp.) capable of processing TCP as a sole source of C and energy was reported in 1997 (Feng et al., 1997), and a recent study by Li et al. (2010) reported *Ralstonia* sp. to degrade concentrations of TCP as a sole source of C and energy. However, little literature is available on the microbial metabolism of TCP as a nitrogen (N) source. Although a significant phylogenetic diversity of microorganisms capable of degrading OP pesticides and their metabolites have been isolated, the potential for degradation is highly influenced by biotic and abiotic environmental factors (Mrozik and Piotrowska-Seget, 2010; Niti et al., 2013). Therefore, it has been highlighted that the characterization of more diverse groups of biodegrading microbial isolates will increase the flexibility of the development of effective bioremediation technologies tailored to local environmental conditions (e.g., pH, nutrients, and soil moisture; Singh, 2010). More broadly, it will improve our understanding of the metabolic routes through which biodegradation of these compounds can take place (Singh and Macdonald, 2010).

The present study aimed to isolate bacterial strains, from sugarcane farm soil, able to rapidly and efficiently degrade both CP and TCP. The isolation of indigenous bacterial strains capable of metabolizing CP and TCP, for *in situ* bioremediation, is favorable given that they are well adapted to the local conditions. In addition to this, the study also aimed to identify the mode of biodegradation (mineralization or co-metabolism) and nutritional requirements of the isolates, in order to provide a more comprehensive knowledge for the future use of microbial isolates in bioremediation. Through repetitive enrichment and successive culturing, indigenous bacterial strains were examined for their potential to degrade CP and TCP (alone and together) in liquid media under various nutritional conditions.

## Materials and Methods

### Site Description and Sample Collection

Soil was retrieved from five sugarcane farms in the Mackay, Burdekin, and Tully areas in Queensland, Australia, with a history of pesticide use to control sugarcane grub. Three out of five sites were located in the Burdekin district (site 1, 2, and 4). Site 3 was located in the Mackay district and site 5 in the Tully region. The history of pesticide application differed between all the sites in terms of number of pesticide applications, amount applied and years applied. Soils from the sites Burdekin and Tully that received annual field application of CP developed an enhanced rate of CP degradation 13 years ago (Supplementary Table 1). The use of CP was discontinued due to a lack of efficacy against the target pests. On the other hand, CP is still effectively used to control pests at Mackay. The soil type and sugarcane grub pesticide history for each site is given in Supplementary Table 1.

Sampling was undertaken within and outside (headland, i.e., no farming) sugarcane farms at each site. Sampling from within the farm was taken from the row at least 8 m in from the headland. Samples from the outside were taken from the headland and surrounding areas, approximately 6–8 m from the farm of sugarcane. Sampling was avoided downhill from the fields to prevent contamination of active ingredients with runoff. Within each site, three replicate soil samples were collected from within the farm (designated R) and headland (designated H). Each replicate was composed of three soil sub-samples taken by digging a hole about 30 cm deep with a shovel. These three sub-samples were mixed together and a composite 2 kg sample was taken for each replicate. The soils were kept at 4^o^C in the dark until further use. Fresh soil samples were sieved through a 2 mm sieve to separate vegetation and other coarse particles from the soil and analyzed for total C and N content, pH, and moisture. For determining soils total C and N, the samples were milled into a fine powder by using a Retsch mill at a frequency of 20 Hz for 2 min. Total C and N content was then quantified with a CHN analyzer according to the manufacturer’s instructions (Leco TruSpec Micro, USA). pH was determined in a 1:2.5 (wt/vol.) diluted water suspension and measured with a pH meter (Mettler Toledo, Australia). Soil moisture content was assessed by measuring the difference of weight of soil samples before and after oven drying at 105°C for 24 h, and expressed as percentage of soil weight. Soil properties for each of the soil types for each of the sugarcane farms are summarized in **Table 1**.

**Table 1:** Soil properties measured at the five sites (sugarcane farms).

Site name	Soil type	pH (±SE)	%Carbon (±SE)	%Nitrogen (±SE)	%Moisture (±SE)
Burdekin I	1R	6.8 ± 0.1	1.12 ± 0.2	0.05 ± 0.0	1.88 ± 0.2
	1H	7.0 ± 0.1	1.27 ± 0.1	0.02 ± 0.0	1.95 ± 0.1
Burdekin II	2R	6.5 ± 0.0	1.49 ± 0.1	0.08 ± 0.0	1.96 ± 0.0
	2H	6.6 ± 0.0	0.88 ± 0.04	0.06 ± 0.0	1.53 ± 0.0
Mackay	3R	5.1 ± 0.1	1.85 ± 0.1	0.08 ± 0.0	2.24 ± 0.1
	3H	5.7 ± 0.0	2.36 ± 0.0	0.02 ± 0.0	2.35 ± 0.0
Burdekin III	4R	6.5 ± 0.1	1.18 ± 0.1	0.06 ± 0.0	1.85 ± 0.1
	4H	6.9 ± 0.1	1.05 ± 0.1	0.06 ± 0.0	2.26 ± 0.3
Tully	5R	6.4 ± 0.1	0.96 ± 0.0	0.04 ± 0.0	1.04 ± 0.0
	5H	5.0 ± 0.2	0.87 ± 0.0	0.05 ± 0.0	1.09 ± 0.0

*Carbon (%) and Nitrogen (%) represents the total carbon and nitrogen of the soil sample. Each data point represents the mean of three experimental replicates (*n* = 3). Here, H represents the headland area with no past history of pesticide application and R represents the farm site with past history of pesticide application.*

### Soil Incubations

A commercial formulation of CP 500 EC (500 g/L, Nufarm) was used in this study to investigate which soil types were able to degrade CP at a rapid rate. About 250 g of soil (*n* = 3 for both R and H types) from all the five sites were placed into plastic jars and mixed with a solution of CP to a final concentration of 10 mg/kg. Soils treated with the pesticide were left for 3–4 h in a fume hood for drying. The water holding capacity of the soil was adjusted to 40% and was maintained by regular addition of Milli Q water. The screw cap plastic jars containing the treated soil were incubated in the dark at room temperature. All the soil-pesticide combinations were sampled periodically up to 105 days to determine the microbial properties and degradation of pesticide. After 45 days or when more than 75% of the initial concentration of CP disappeared, another spike of the pesticide was applied to reach a final concentration of 10 mg/kg. The soils were retreated with a third application of pesticide (10 mg/kg) on day 50 after the second treatment, when maximum degradation of pesticide took place.

At regular intervals, pesticides and their metabolites were extracted from soil (2.5 g) by mixing with acetonitrile:water (90:10, 5 mL) in glass vials. The vials were vortexed and the pesticide extraction was done by shaking the mixture for 1 h on a shaker (130 rpm). The samples were centrifuged for 5 min at 15,000 rpm and the supernatant was filter sterilized through a 0.22-μm nylon syringe filter for high-performance liquid chromatography (HPLC) analysis using an Agilent 1260 Infinity HPLC system. CP and its metabolite, TCP, were separated on an Agilent Poroshell 120 column (4.6 × 50 mm, 2.7 μm) with Agilent ZORBAX Eclipse Plus-C18 guard column (4.6 × 12.5 mm, 5 μm). The injection volume was 10 μL and the mobile phase was acetonitrile:water (75:25), acidified with 1% phosphoric acid. The analytes were eluted at 40°C with an isocratic mobile phase flow rate of 0.8 mL/min for 4.5 min. The pesticides and their metabolites were detected spectrophotometrically at 230 nm.

### Enrichment of CP and TCP Degraders

The soil representing samples 4H, 4R, and 5R was subsequently used for the enrichment of potential CP and TCP degraders because they showed enhanced degradation of CP in the laboratory-based degradation study mentioned above. Of these soils (4H, 4R, and 5R), one soil (5R) was further treated separately with TCP (10 mg/kg) to enrich for TCP degraders. The enrichment of CP/TCP degraders was carried out in liquid media with degradation of pesticide monitored every 2 days. Pesticides and their metabolites were extracted from liquid culture (1 mL) by mixing with 100% acetonitrile (5 mL), vortexed followed by shaking the mixture for 1 h on a shaker (130 rpm). The samples were centrifuged for 10 min at 15,000 rpm, after which a sub-samples of the clear supernatant was analyzed directly by HPLC. The HPLC conditions used were the same as those described earlier.

Two different media, a mineral salt medium (MSM) supplemented with nitrogen and a soil extract medium (SEM) were used for liquid enrichments and isolation of CP and TCP degraders. The composition of media and preparation methods have been described in detail elsewhere (Cullington and Walker, 1999; Karpouzas et al., 2000). The C-source was provided in the form of formulated CP or TCP dissolved in an organic solvent (20 mg/mL in methanol) in glassware and methanol was allowed to be evaporated. Then autoclaved MSM and SEM were added aseptically to achieve a concentration of 20 mg/L. The pH of both media (MSM and SEM) was maintained at 7.0 ± 0.2 using sulfuric acid to allow for the growth of indigenous degraders of pesticide present in the pesticide-treated soils. MSM agar and SEM agar containing CP or TCP were prepared in a similar way, except that agar was added (15 g/L).

### Isolation of CP and TCP Degraders

The isolation of CP degraders from 4R and 4H (only CP-spiked soil samples) and TCP degraders from 4R and 4H (only CP-spiked soil samples) was attempted. These variations were set up to screen for strains that could degrade CP and/or TCP in liquid enrichment medium alone or together. Approximately 1.5 g of soil (wet weight) from each replicate was added to 10 mL of MSM with sterile glass beads and vortexed for 1 min. About 2 mL of the supernatant was then used to inoculate separate bottles containing 40 mL of MSM and 20 mg/L of CP or TCP. All replicates of each soil type and treatment were treated separately for isolation purposes. Pesticide degraders were enriched in the dark on a shaker at 180 rpm for 4 days at 28°C. A second enrichment was done by transferring 1 mL of pre-grown culture from each replicate to the fresh respective media (40 mL) and the same incubation conditions were followed for another 4 days. Appropriate un-inoculated sterile controls were also maintained throughout the experiment (Singh et al., 2004).

Following the second enrichment, a 10 × 10-fold dilution series was prepared and 0.1 mL of each dilution was spread in triplicates on MSM agar plates (with 20 mg/L CP or TCP concentration) and incubated for 2 days at 28°C. Purity streaking for isolated colonies was done on SEM agar plates.

### Colony DNA Extraction and Amplification by Polymerase Chain Reaction (PCR)

Well-isolated bacterial cells (from soil cultures) were picked and carefully resuspended in 10 μL of nuclease-free water. DNA was extracted by boiling the cells at 95°C for 3 min and then cooling on ice for 1 min. The resuspended mix was then centrifuged for 5 min at 15,000 rpm. 1 μL of the supernatant was directly used for PCR amplification, targeting the bacterial 16S *rRNA*. The primer set used for the amplification of 16S *rRNA* gene was 27f (5′-AGAGTTTGATCMTGGCTCAG-3′) and 1492r (5′-TACGGYTACCTTGTTACGACTT-3′; Lane, 1991; Dees and Ghiorse, 2001; Martin-Laurent et al., 2001). This primer set amplifies a >1400 bp region. The PCR conditions used were 0.5 μL of each primer (20 pmol), 1 μL of poly-deoxynucleoside triphosphate mix (dNTPs, 20 mM), 5 μL 10× NH_4_^+^ PCR buffer, 2 μL of MgCl_2_ (50 mM), 1 μL BSA (20 mg/mL), 0.5 μL of Taq polymerase (all reagents from Bioline, USA), and 38.5 μL of sterile nuclease-free water to make the reaction volume 50 μL. Negative controls, with 1 μL of molecular grade water as a template, were included in all sets of PCR reactions to provide a contamination check. All amplifications were carried out in a DNA Engine Dyad^®^ Peltier Thermal Cycler (Bio-Rad, USA). The PCR protocol began with a denaturation step at 95°C for 4 min followed by 29 cycles of denaturation at 94°C for 30 s, annealing at 55°C for 1 min and extension at 72°C for 1 min. The protocol was concluded with an additional final extension at 72°C for 10 min. After amplification, 3 μL of PCR products were electrophoretically separated and visualised in 1× TBE, 1% (w/v) agarose gel stained with SYBR^®^ Safe (Life technologies, USA) to confirm the amplification and specificity. The DNA amplicons were then purified using Wizard^®^ SV Gel and PCR clean-up system as per the recommendation by the supplier (Promega Corporation, USA) and quantified with NanoDrop 2000C spectrophotometer (Thermo Fisher, USA) before submitting samples for sequencing.

### Sequencing

Purified PCR products were sequenced using the BigDye Terminator v3.1 kit (Applied Biosystems). About 15 ng of DNA template and 3 pmol of both forward (27f) and reverse primer (1492r) were used per 20 μL reaction mixture in separate reactions. 16S *rRNA* gene was amplified with the above primer set resulting in product length of ∼1400 bp. The amplified PCR products were collected, purified and sequenced. The quality of the sequence was checked using Sequencher 4.10.1 (Gene Codes Corporation) where both forward and reverse strands were edited, assembled and aligned together to generate a contig for further analyzes.

All 16S *rRNA* partial sequences (>1200 bp) isolates were compared with those available on a BLAST search of the GenBank database^1^. Sequences with the greatest similarity were extracted and aligned with MUSCLE (MEGA 6). Those aligned DNA sequences were then used to construct a phylogenetic tree using MEGA 6 (Molecular Evolutionary Genetics Analysis; Tamura et al., 2013) by performing the maximum likelihood tree analysis with 1000 bootstrap replicates using Kimura-2-parameter model with Gamma distribution (K2 + G). All sequences have been submitted to National Centre for Biotechnology Information (NCBI) database (accession number: KY646471–KY646476).

### Degradation of CP and TCP as a Source of Carbon (C), Nitrogen (N), and Phosphorus (P)

The capacity of the isolates to degrade CP and TCP as C-, N- and P-source was determined. For this purpose, MSM was modified and adapted from Coleman et al. (2002). The medium was supplemented with CP or TCP (20 mg/L). The following solutions/components were used to make up the media (g/L MQ water): Solution-1: 2.27 g K_2_HPO_4_, 0.95 g KH_2_PO_4_, 0.67 g (NH_4_)2SO_4_; A mixture of vitamin Supplement (purchased from ATCC^®^, USA); Trace salt solution (Solution 2): 6.37 g Na_2_EDTA.2H_2_O, 1.0 g ZnSO_4_.7H_2_O, 0.5 g CaCl_2_.2H_2_O, 2.5 g FeSO_4_.7H_2_O, 0.1 g NaMoO_4_.2H_2_O, 0.1 g CuSO_4_.5H_2_O, 0.2 g CoCl_2_.6H_2_O, 0.52 g MnSO_4_.H_2_O, and 29.30 g MgSO_4_. pH of the trace salt solution was adjusted to 6.4 ± 0.2, which is very critical for all the trace salts to dissolve and form homogenous solution. The solution was filter-sterilized (0.22 μm pore size, Millipore) and stored at 4°C in a foil-wrapped bottle.

For preparation of media, vitamin supplement (0.5% v/v) and trace salt solution (0.2% v/v) were added aseptically to solution-1 after autoclaving, to avoid precipitation of salts. Four variations of medium were set up: CP/TCP as a sole source of C (+N + P – C); CP/TCP as a sole source of N (–N + P + C); CP/TCP as a source of P (+N – P + C) and CP/TCP as the sole C and N source (–N + P – C). Bulk media were autoclaved and 10 mM glucose (C_6_H_12_O_6_) was added as an alternative C-source where required. The media without N was prepared simply by omitting ammonium sulfate [(NH_4_)_2_SO_4_] and for P-free media K_2_HPO_4_ and KH_2_PO_4_ were replaced with 20 mM 3-(*N*-morpholino)propanesulfonic acid (MOPS). No glucose or ammonium sulfate was added to prepare a C- and N-free media. The final pH of the media was adjusted to 7.0 ± 0.2.

The bacterial isolates were first grown in SEM supplemented with CP or TCP (20 mg/L) to get an increased active biomass of the degrading culture before inoculating them into MSM with different nutrient compositions. The cultures were centrifuged at 14,000 rpm for 5 min. The supernatant was discarded and the pellet was then washed three times and resuspended in autoclaved tap water before inoculating in different media. Media with different compositions (in terms of C, N, and P) were used to understand the physiology and kinetics of degrading bacteria. Triplicate samples (20 mL) of each medium were inoculated with actively growing isolates of CP or TCP degraders (1 mL) to a final optical density between 0.01 and 0.03 at 600 nm (OD_600_) based on the turbidity of the cell suspension. Triplicate sets of each media combination without inoculum were maintained in all the experiments as controls.

### Statistical Analyzes

To investigate the differences in the pesticide degradation between different isolates, an independent samples *t*-test was carried out, with the significance level (*p* value) set at 0.05. The degradation of the pesticides was ascribed by the first-order function (*C*_t_ = *C*_o_ × e^-kt^). The rate constant, *k*, was estimated by plotting ln*C* over time with –*k* being equal to the slope of the linear regression representing this relationship; hence, *k* is an estimate of the average rate constant across all sampling points for each sample. The half-lives of the pesticides were obtained by function *t*_1/2_ = ln2/*k*. Statistical analyses were carried out with the software package IBM SPSS Statistics 17 (SPSS, Inc.).

## Results

### Degradation of CP and TCP in Soil and MSM Medium

**FIGURE 1:**
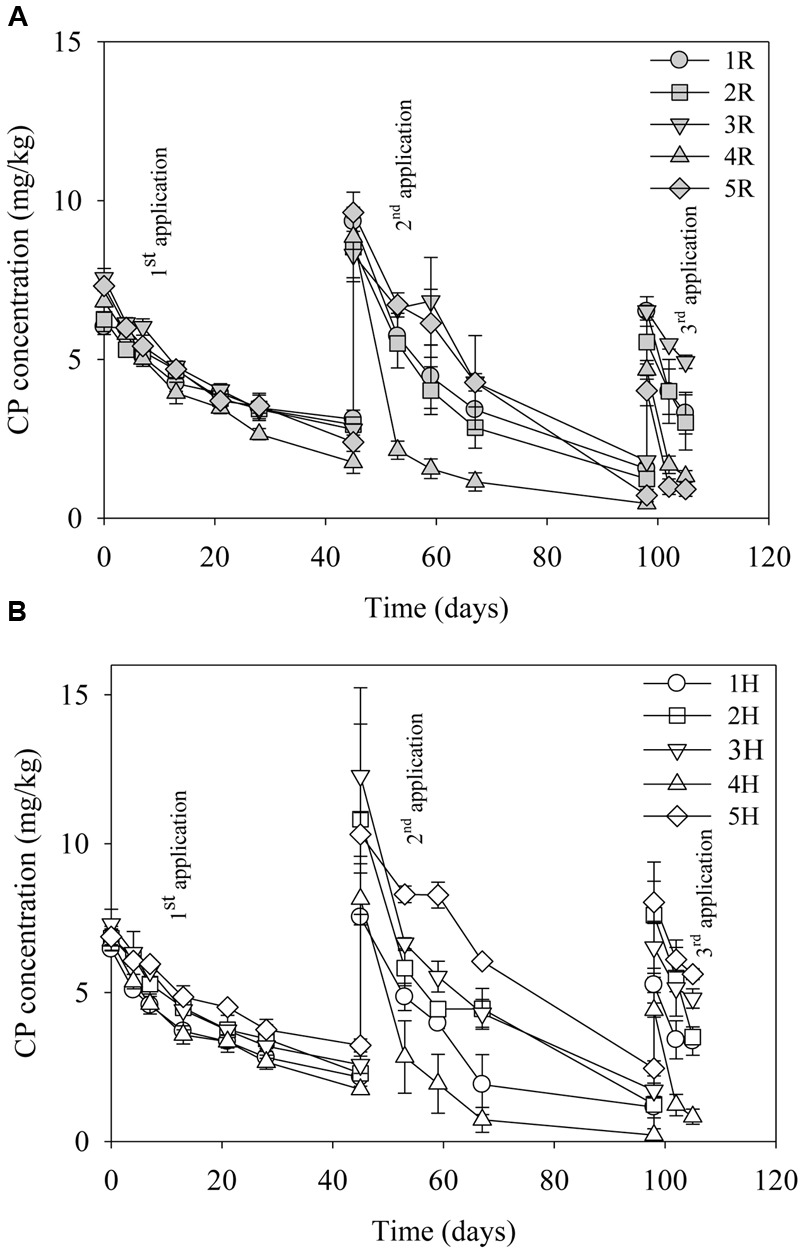
**Degradation of chlorpyrifos (CP) in the different sites (soils) after three repeated laboratory applications (10 mg/kg). (A)** Sites treated with pesticides in the fields (R) and **(B)** headland sites not treated with pesticides (H). Error bars represent standard error of the mean (*n* = 3). No errors bars are of the same size or smaller than the plot symbols.

The degradation pattern of CP after repeated CP applications to the same soil is shown in **Figures 1A,B**. The rate constant, *k*, and thus the half-life of CP differed between test sites. The kinetic data of CP degradation of all the sites is given in **Table 2**. The results indicate a general decrease in the half-life of CP during the sequential applications; therefore, suggesting enhanced degradation of the compound at all the test sites except for site 3 (Mackay) where half-life of CP remained similar after second and third applications. Among the sites, the disappearance of CP after three applications was particularly rapid in 4H, 4R, 5H, and 5R. However, the rate constant and half-life calculated following the third CP application should be interpreted with care given that this is based on only three sampling points.

**Table 2:** Kinetics data of chlorpyrifos (CP) at five different sites following multiple applications of CP to the same soil type.

Site	Soil type	Application	*C*_0_	Est. *C*_0_	Rate constant, *k* (day^-1^)	Half-life, *t*_1/2_ (days)	*R*^2^
Burdekin I	1R	1	6.02	5.75	0.016	44.43	0.904
		2	9.34	7.55	0.024	28.88	0.932
		3	6.51	6.31	0.040	17.20	0.966
	1H	1	6.45	5.58	0.023	30.27	0.947
		2	7.53	6.07	0.025	27.73	0.862
		3	5.26	5.01	0.027	26.06	0.835
Burdekin II	2R	1	6.26	5.81	0.017	41.51	0.951
		2	8.53	7.01	0.026	26.76	0.935
		3	5.55	5.56	0.035	19.86	1.000
	2H	1	6.82	6.50	0.024	29.25	0.983
		2	10.82	8.66	0.028	24.41	0.937
		3	7.60	7.82	0.046	15.20	0.976
Mackay	3R	1	7.56	6.82	0.022	31.65	0.953
		2	8.30	8.49	0.023	30.01	0.972
		3	6.52	6.50	0.017	40.77	0.997
	3H	1	7.30	6.65	0.024	29.50	0.947
		2	12.28	9.62	0.027	25.39	0.934
		3	6.52	6.41	0.019	36.67	0.950
Burdekin III	4R	1	6.82	6.35	0.030	23.42	0.985
		2	8.86	4.25	0.029	24.24	0.659
		3	4.67	4.30	0.065	10.60	0.918
	4H	1	6.75	5.94	0.028	24.58	0.966
		2	8.14	4.62	0.034	20.63	0.779
		3	4.41	4.01	0.079	8.76	0.927
Tully	5R	1	7.31	6.62	0.024	29.50	0.972
		2	9.62	9.80	0.034	20.27	0.995
		3	4.02	3.51	0.072	9.69	0.845
	5H	1	6.88	6.50	0.017	40.77	0.958
		2	10.31	10.63	0.023	30.67	0.987
		3	8.03	7.88	0.023	9.69	0.952

*The degradation rate constant, *k*, was derived by plotting ln*C* over time with –*k* being equal to the slope of the linear regression fitted to this data. *C*_*0*_ represents measured CP concentrations at time 0 for each pesticide application, Est. *C*_*0*_ represents the estimated CP concentration based on the linear regression of ln*C* over time and the *R*^*2*^-values are presented as a measure of how well the linear regression model fit the data. The half-lives of the pesticides were obtained by function *t*_*1/2*_ = ln2/*k*. H represents the headland area with no past history of pesticide application and R represents the farm site with past history of pesticide application.*

**FIGURE 2:**
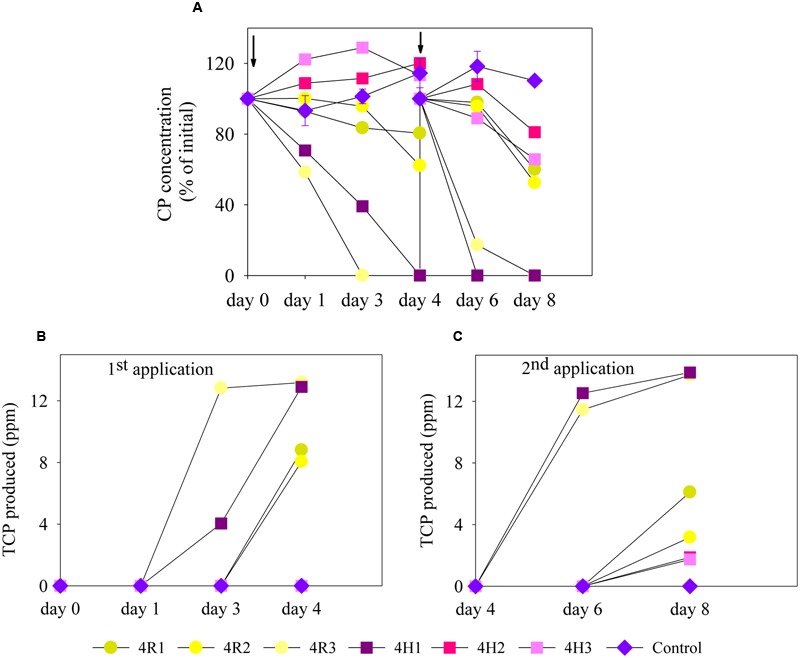
**Degradation of CP and production of TCP following two successive applications of CP in mineral salt medium (MSM) inoculated with bacterial communities (4R and 4H) and control samples; (A)** CP degradation, **(B)** TCP production after the first CP application (enrichment), and **(C)** TCP production after the second CP application (enrichment). For visualization purposes, all the replicates of 4R and 4H were plotted individually here. Error bars on control (un-inoculated treatment) represents standard error of the mean (*n* = 3). No visible error bar means that bars are of the same size or smaller than the plot symbols. The arrows on the graph **(A)** represents the day when re-spiking was done. TCP, 3,5,6-trichloro-2-pyridinol.

Degradation of CP in MSM (liquid enrichment media) supplemented with soil microbial inoculum from 4R (4R1, 4R2, and 4R3) and 4H (4H1, 4H2, and 4H3) soils are shown in **Figure 2A**. For the first CP application (liquid enrichment), CP degradation rate occurred more rapidly in 4R3 and in 4H1 as complete degradation of CP took place in just 3 and 4 days, respectively. After 4 days of incubation, soil samples 4R1 and 4R2 showed only 20 and 38% CP degradation, respectively, whereas no degradation was seen with microbial inoculum from 4H2 and 4H3. The primary metabolite of CP, TCP, was found in all samples displaying CP degradation (**Figure 2B**).

A second application of CP further increased the degradation rate for both 4R3 and 4H1, with most CP degraded in 2 days. Degradation also started earlier for other soil samples (4R1 and 4R2). The second enrichment also led to the degradation of CP in 4H2 and 4H3, degrading 20 and 30% of CP, respectively. TCP was also produced following a second application of CP due to CP degradation (**Figure 2C**). However, no degradation of accumulated TCP was seen in any of the soil samples. No significant degradation of CP was seen in any of the un-inoculated controls as shown in **Figure 2A**. It was surprising to see that one of the replicates from both soil types showed an accelerated degradation during first application. However, all replicates (for both 4R and 4H) showed an increased rate of degradation after the second application, likely due to increase in the population size of microbial CP degraders. As this study’s primary aim was to isolate potential CP and/or TCP degraders, the replicates that showed enhanced degradation (4R3 and 4H1) in MSM liquid media were used for isolation purpose.

### Isolation of CP and TCP Degraders

Various attempts to isolate single colonies with CP-degrading abilities from enriched cultures were made. A total of six potential degraders were obtained after successive sub-culturing from 4R3 and 4H1 soils and named 4H1-M1, 4H1-M2, 4H1-M3, 4H1-M4, 4R3-M1, and 4R3-M2. No fungal growth was seen in any of the agar plates (data not shown). By contrast, no detectable degradation of TCP was seen with any of the soil types (4H, 4R, and 5R) when added externally in MSM (data not shown). There was no effect of repeated transfer on TCP degradation rates. Despite no degradation of added TCP in liquid MSM media, the microbial inoculum from soils enrichments of 4H, 4R, and 5R were plated on MSM agar plates to check for their ability to grow in presence of added TCP on solid media. However, microbial inoculum failed to grow from any of the enriched soils (4H, 4R, and 5R) on MSM agar when TCP was added externally, during the set incubation time. Thus, they were dropped from further analyzes and only the CP-degrading isolated bacterial strains (4H1-M1, 4H1-M2, 4H1-M3, 4H1-M4, 4R3-M1, and 4R3-M2) were further analyzed for their ability to degrade (i) CP, (ii) TCP, as an accumulated metabolite, and (iii) TCP, when added externally.

### Molecular Characterization of Isolated CP and TCP Degraders

To assess the phylogeny of the isolates, a BLASTN analysis (16S *rRNA* sequence) was carried out through GenBank^2^. This showed that all six the isolates clustered into the Proteobacteria group, but belonged to four different genera. The 16S *rRNA* sequence of the isolate 4R3-M1 illustrated a high similarity to the reference sequence from members of the genus *Xanthomonas*, showing 99% similarity with *Xanthomonas* sp. R9-741 (GenBank Accession no. JQ660016), *Xanthomonas* sp. RP-B14 (GenBank Accession no. FM997990), *Xanthomonas* sp. 33DCP (GenBank Accession no. HQ891021), *Xanthomonas campestris* (GenBank Accession no. NR074936), and *Xanthomonas* sp. Y4 (GenBank Accession no. KC708559). Strain 4H1–M3 clustered with genus *Pseudomonas* showing 100% similarity with *Pseudomonas putida* (GenBank Accession no. KF815695, KC189961, and GQ200822), *P. plecoglossicida* BF-1 (GenBank Accession no. FJ592171) and other *Pseudomonas* species (GenBank Accession no. KJ733977, HM748053, EU851056, EU375660, and DQ079062). Isolate 4H1–M2 clustered distantly with the clade of genus *Lysobacter* showing 99% similarity with uncultured bacteria (GenBank Accession no. DQ404715, JX236701, and AB234277) and *Lysobacter* sp. ITP09 (GenBank Accession no. FR667176).

**FIGURE 3:**
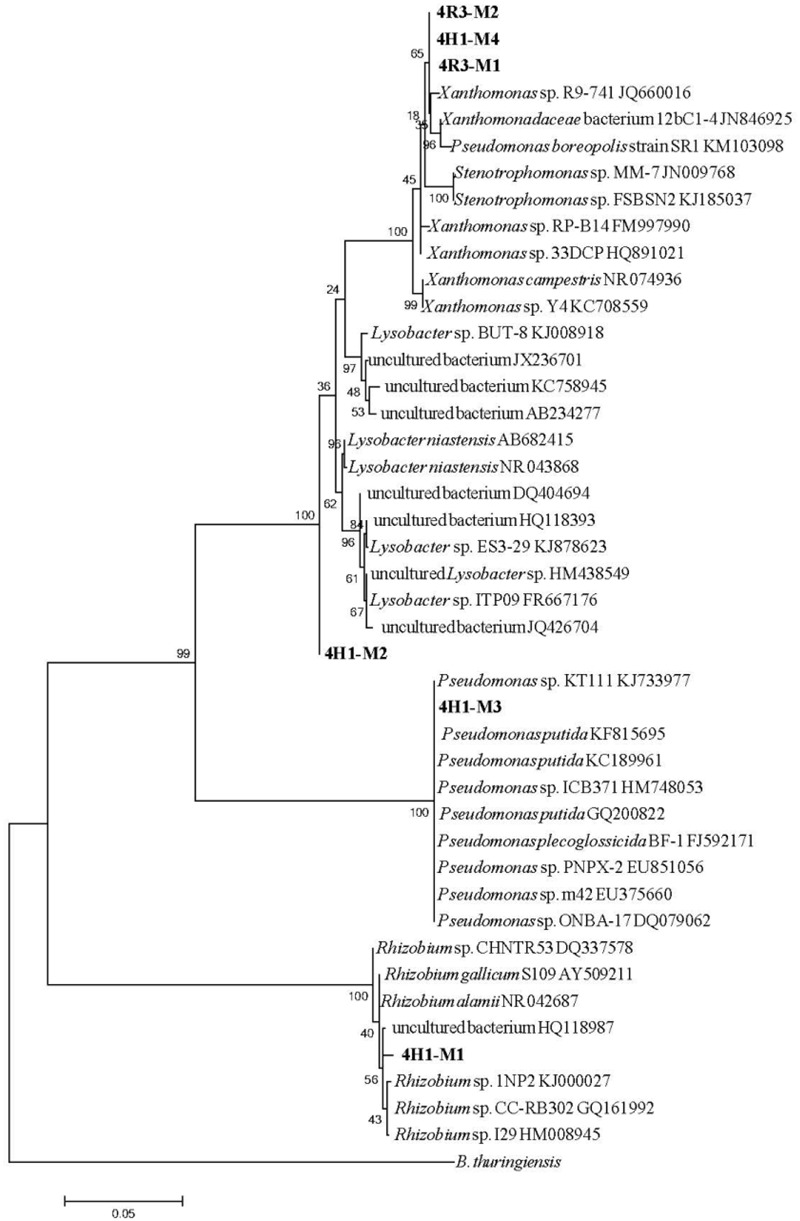
**Maximum likelihood tree showing the phylogenetic relationships between all six isolates (in bold) and related species based on the 16S rRNA gene sequences retrieved from NCBI.** The tree was rooted with *Bacillus thuringiensis.* Bootstrap values are expressed as randomization of 1000. The scale bar represents the evolutionary distance of 0.05.

Isolate 4H1–M1 was related to genus *Rhizobium* and showed 99% similarity with *Rhizobium gallicum S*109 (GenBank Accession no. AY509211), *Rhizobium alamii* (GenBank Accession no. NR042687), *Rhizobium* sp. CHNTR53 (GenBank Accession no. DQ337578), *Rhizobium* sp. 1NP2 (GenBank Accession no. KJ000027), *Rhizobium* sp. CC-RB302 (GenBank Accession no. GQ161992), and *Rhizobium* sp. I29 (GenBank Accession no. HM008945). A phylogenetic tree that depicts the position of all the isolates and their related species is presented in **Figure 3**. Based on these observations, the strains were designated as *Xanthomonas* sp. 4R3-M1, *Pseudomonas* sp. 4H1-M3, *isolate* sp. 4H1-M2 and *Rhizobium* sp. 4H1-M1.

### Degradation of CP and TCP as a Source of Carbon (C), Nitrogen (N), and Phosphorus (P)

**FIGURE 4:**
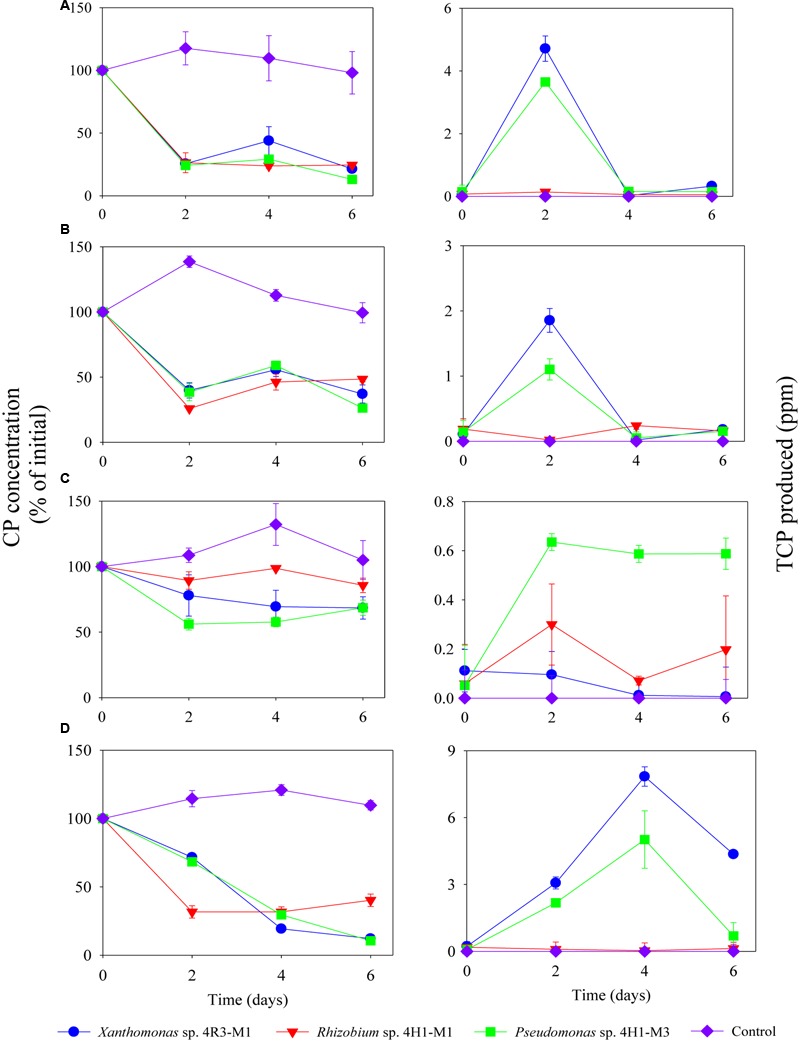
**Degradation of CP (%) and production of TCP (ppm) by *Xanthomonas* sp. 4R3-M1, *Rhizobium* sp. 4H1-M1 and *Pseudomonas* sp. 4H1-M3 in different media: (A)** MSM (+N+ P - C), **(B)** MSM (-N + P + C), **(C)** MSM (+N - P + C) and **(D)** SEM. Error bars represent standard error of the mean (*n* = 3). No visible error bar means that bars are of the same size or smaller than the plot symbols.

**FIGURE 5:**
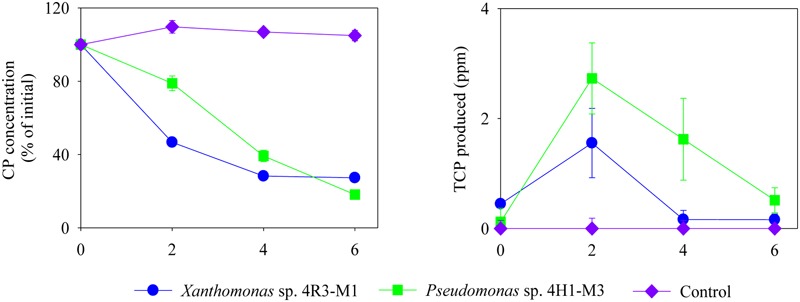
**Degradation of CP (%) and production of TCP (ppm) by *Xanthomonas* sp. 4R3-M1 and *Pseudomonas* sp. 4H1-M3 in MSM (-N + P - C).** Error bars represent standard error of the mean (*n* = 3). No visible error bar means that the bars are of the same size or smaller than the plot symbols.

Isolates representing three bacterial families (*Xanthomonas* sp. 4R3-M1, *Pseudomonas* sp. 4H1-M3, and *Rhizobium* sp. 4H1-M1) were selected for further CP and TCP degradation and were screened to determine their ability to degrade CP in SEM and MSM with different nutrient compositions (+N + P - C, -N + P + C, +N – P + C and -N + P - C) (**Figures 4A–D, 5**). The CP-degradation ability of all the strains was greater in the media MSM without C (+N + P - C; *p* < 0.05; **Figure 4A**), MSM without N (-N + P + C; *p* < 0.05; **Figure 4B**) and SEM (*p* < 0.05; **Figure 4D**) when compared to MSM without P (+N – P + C; *p* > 0.05; **Figure 4C**).

The strains *Xanthomonas* sp. 4R3-M1, *Pseudomonas* sp. 4H1-M3, and *Rhizobium* sp. 4H1-M1 utilized 80, 90 and 75% of CP in MSM (+N + P - C), respectively, in 6 days of incubation. About 90% CP degradation was observed in SEM when inoculated with *Xanthomonas* sp. 4R3-M1 and *Pseudomonas* sp. 4H1-M3 (**Figure 4D**). By contrast, the degradation potential of *Rhizobium* sp. 4H1-M1 was lower, utilizing only 60% of the CP within 6 days of incubation.

The lowest degradation rates of CP were observed in MSM (-N + P + C; **Figure 4B**) for all bacterial strains (*Xanthomonas* sp. 4R3-M1: 60%, *Pseudomonas* sp. 4H1-M3: 70%, and *Rhizobium* sp. 4H1-M1: 50%) when compared to MSM (+N + P - C) and SEM (**Figures 4A,D**, respectively). TCP accumulated at similar rate as that of decrease in CP concentration (**Figures 4A–D**). TCP yields for *Xanthomonas* sp. 4R3-*M1* and *Pseudomonas* sp. 4H1-M3 were highest in SEM (**Figure 4D**) followed by MSM (+N + P - C; **Figure 4A**), and MSM (-N + P + C; **Figure 4B**). No TCP degradation was observed during initial accumulation, which may be considered a lag phase required to induce the production of TCP-degrading enzymes. However, CP degradation by *Rhizobium* sp. 4H1-M1 did not result in the production/accumulation of TCP in any of the media, which was not consistent with other two strains. Conversely, TCP produced as a result of CP degradation by *Xanthomonas* sp. 4R3-M1 and *Pseudomonas* sp. 4H1-M3 was also degraded in all media types (**Figure 4**). It was difficult to conclude TCP utilization in MSM (+N – P + C), because CP degradation was reduced in this media resulting in lower TCP production (**Figure 4C**).

No degradation of CP or production of TCP was seen in any of the un-inoculated controls (**Figure 4**). From the above results, *Xanthomonas* sp. 4R3-M1 and *Pseudomonas* sp. 4H1-M3 seemed to be versatile CP degraders and were used to study the effect on CP degradation in MSM (-N + P - C; i.e., without carbon and nitrogen). *Xanthomonas* sp. 4R3-M1 and *Pseudomonas* sp. 4H1-M3 showed similar degradation patterns utilizing 70 and 80% of CP, respectively. TCP was also consumed in the process (**Figure 5**).

**FIGURE 6:**
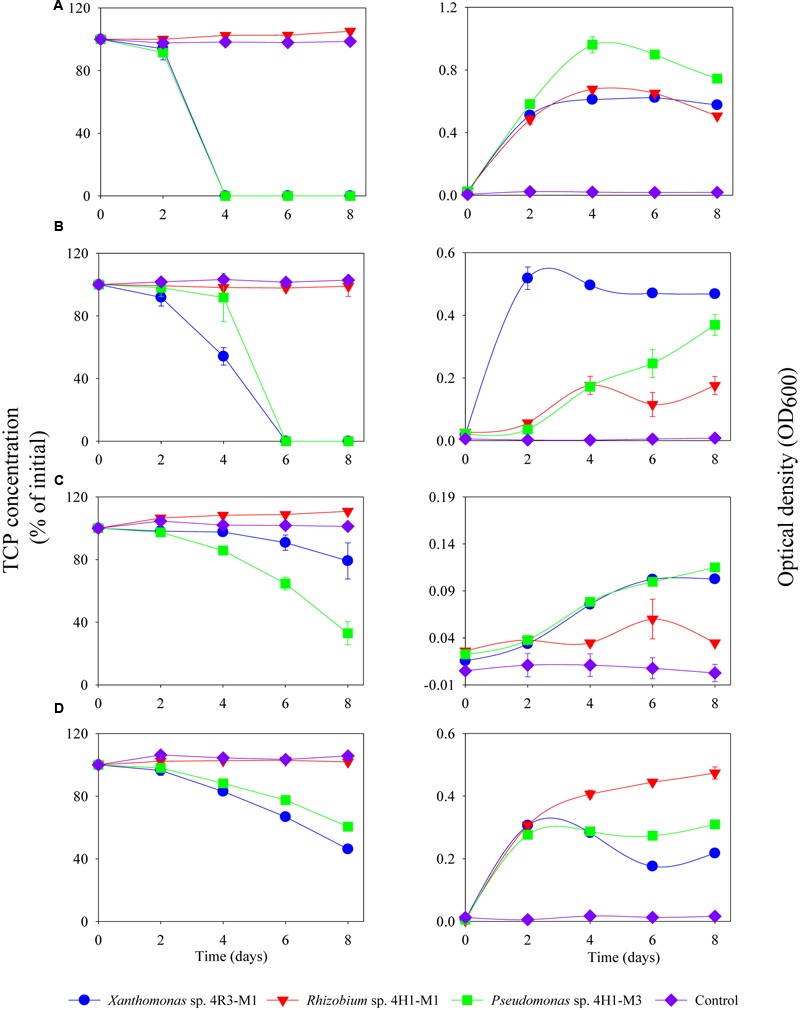
**Degradation of TCP (%), externally added, and corresponding growth (OD_600_) of *Xanthomonas* sp. 4R3-M1, *Rhizobium* sp. 4H1-M1 and *Pseudomonas* sp. 4H1-M3 in different media: (A)** MSM (+N + P - C), **(B)** MSM (-N + P + C), **(C)** MSM (+N - P + C), and **(D)** SEM. Error bars represent standard error of the mean (*n* = 3). No visible error bar means that the bars are of the same size or smaller than the plot symbols.

**FIGURE 7:**
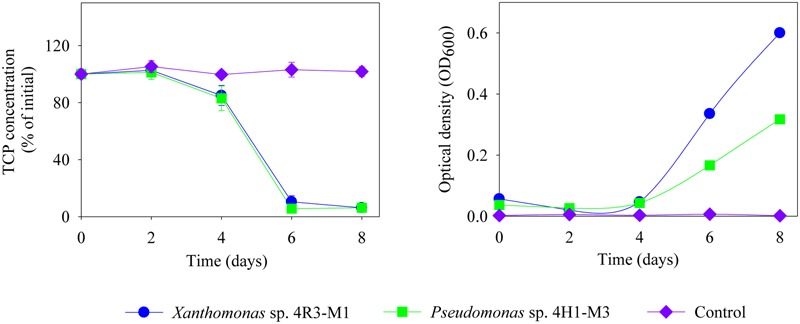
**Degradation of TCP (%), externally added and corresponding OD_600_ of *Xanthomonas* sp. 4R3-M1 and *Pseudomonas* sp. 4H1-M3 in MSM (-N + P - C).** Error bars represent standard error of the mean (*n* = 3). No visible error bar means that the bars are of the same size or smaller than the plot symbols.

The TCP degradation pattern by all three strains in different media composition is shown in **Figure 6**. The degradation dynamics of TCP, in different media compositions, were almost similar for *Xanthomonas* sp. 4R3-M1 and *Pseudomonas* sp. 4H1-M3. Both strains could degrade 100% TCP in MSM (+N + P - C; *p* < 0.001) as a C-source within 4 days and MSM (-N + P + C; *p* < 0.001) as N-source within 6 days (**Figures 6A,B**, respectively). After 8 days of incubation, TCP utilization for *Xanthomonas* sp. 4R3-M1 was 20% (not statistically significant from control) (MSMP + N – P + C; *p* > 0.05), 54% (SEM; *p* < 0.01), and that of *Pseudomonas* sp. 4H1-M3 was 67% (MSM+N-P+C; *p* < 0.05) and 40% (SEM; *p* < 0.01) (**Figures 6C,D**, respectively). The degradation of TCP was slower in MSM (+N – P + C) and SEM showing an effect of media on degradation. In all cases, TCP degradation was accompanied by an increase in bacterial OD_600_. In contrast, *Rhizobium* sp. 4H1-M1 did not degrade TCP in any of the media assessed (*p* > 0.05) and no degradation was observed in un-inoculated controls (**Figure 6**). Since *Rhizobium* sp. 4H1-M1 did not show any TCP degradation when added externally, *Xanthomonas* sp. 4R3-M1 and *Pseudomonas* sp. 4H1-M3 were further studied for their role in TCP degradation in C and N deficient media. Both strains showed similar degradation patterns consuming 90% of TCP in 6 days (*p* < 0.01); however, degradation of TCP by *Xanthomonas* sp. 4R3-M1 was accompanied by a higher bacterial growth (**Figure 7**).

## Discussion

### Isolation of CP and TCP Degraders

In order to isolate potential microorganisms that degrade CP and TCP, we examined the enhanced degradation of these compounds in soils from five sites. Samples from all but one site (i.e., site 3 from the Mackay region) demonstrated enhanced degradation of CP. Other four sites were reported previously for enhanced degradation 13 years ago (Singh et al., 2003a) and use of CP was discontinued due to lack of efficacy against target pest. However, at Mackay, CP is still effectively used and our results suggest that even after repeated applications, enhanced degradation of CP did not establish. Development of enhanced degradation of xenobiotics depends on multiple factors including chemical structure, soil properties, and presence of degrading microbes. This topic is discussed in details by previous works (Karpouzas and Walker, 2000; Singh and Walker, 2006; Singh, 2010 and references within). Our aim was to isolate CP/TCP-degrading microbes from soils which demonstrated rapid degradation of CP and TCP. To achieve this, bacterial consortia were established from selected soils using a selective enrichment technique, providing CP and/or TCP as a sole source of C. When inoculated in MSM (+N – P + C, +N + P - C, and -N + P + C) supplemented with vitamins, isolated bacterial strains were able to degrade CP and/or TCP as a source of energy. Previous studies have reported dehalogenation of chlorine to be an important microbial mechanism for degrading of TCP (Feng et al., 1998; Van Pee and Unversucht, 2003; Math et al., 2010; Cao et al., 2013). Although our understanding of the pathway involved in the microbial metabolism of TCP is still limited, current literature and our results indicate that the bacterial strains attacked the TCP ring structure for C and N only in vitamin-supplemented media. Oxygenases involved in the dehalogenation of the aromatic compounds generally require a reducing co-factor (NADH or NADPH) in addition to Fe (II) and an aerobic environment (Mason and Cammack, 1992). Thus, it is proposed that vitamins might promote the activity of oxygenases/or other basic enzymes responsible for TCP degradation by bacterial isolates in MSM (+N – P + C, +N + P – C, -N + P + C) media. Accordingly, no degradation was seen in the media without vitamins. The above findings provide additional insight into the mechanism of microbial TCP degradation and could be utilized for improving biological degradation of both CP and TCP.

### Molecular Characterization of Isolated CP and TCP Degraders

Different species of *Xanthomonas* and *Pseudomonas* have been reported to degrade OP compounds catabolically as C-, N- or P-sources or co-metabolically (Bano and Musarrat, 2003; Cycoń et al., 2009). However, this is the first time bacteria from the genus *Rhizobium* have been shown to degrade CP and internally formed metabolite TCP. Munnecke and Hsieh (1974) and Tchelet et al. (1993) reported isolates of *Pseudomonas* sp. and *Xanthomonas* sp. that were adapted to grow on parathion by hydrolyzing it. In another recent study, *Xanthomonas* and *Pseudomonas* species were isolated from leaf surfaces in the rape phyllosphere with activity for the biodegradation of dichlorvos, an OP pesticide (Ning et al., 2010). Some *Pseudomonas* sp. alone or in consortia have also shown to degrade CP (Lakshmi et al., 2009; Sasikala et al., 2012) and its primary metabolite TCP (Feng et al., 1997; Lakshmi et al., 2009) in soil and liquid media. Recently, Chawla et al. (2013) isolated *Xanthomonas* sp. that degraded CP. In this study, isolated bacterial strains that belonged to the genus *Xanthomonas* degraded both CP and its primary metabolite TCP simultaneously, a finding that was not reported before.

### Degradation of CP and TCP as a Source of carbon (C), Nitrogen (N), and Phosphorus (P)

One of the important factors that influence the ability of microorganisms to degrade pesticides is the availability of C and nutrients. This study revealed that *Xanthomonas* sp. 4R3-M1, *Rhizobium* sp. 4H1-M1 and *Pseudomonas* sp. 4H1-M3 were able to degrade CP over a wide variation of media composition, including MSM (+N + P – C), MSM (–N + P + C), and MSM (–N + P – C), suggesting that these strains could utilize CP as a source of both C and N. Bacterial species have been reported earlier to utilize OP compounds as a source of C or N (Singh and Walker, 2006). Initial slower rate of CP degradation observed in SEM might be related to soil properties but once the isolates acclimated, the degradation was greater in the SEM than MSM (+N-P+C, +N+P-C, -N+P+C and -N+P-C). This can prove to be important features of these strains for application in bioremediation because SEM is prepared directly from soil it is more similar to real field conditions in terms of nutrient types. These strains utilized CP as a sole source of C and N indicating that the pyridinyl ring of CP had been cleaved and utilized as a source of C for growth and cellular activities.

The degradation ability of all of the three strains was not influenced by the presence of an alternative C-source (glucose) in the MSM (–N + P + C). Karpouzas and Walker (2000) found similar results with ethoprophos degradation by *P. putida*, in which the presence of other C-sources had no effect on the degrading ability of the bacteria. The strains in this study preferred to utilize CP even in nutrient rich media potentially due to the constitutive expression of CP-degrading enzymes even in the presence of readily available C-sources (Anwar et al., 2009; Farhan et al., 2012). This result contrasts with previous findings of Singh et al. (2004) who reported that with addition of C-sources, an *Enterobacter* strain stopped degrading CP and only after 3 days of incubation started utilizing CP again. Further, supplementation of MSM (+N + P – C) with N improved the degradation of CP when compared to MSM (–N + P + C). This finding is supported by earlier studies by Li et al. (2007) and Chawla et al. (2013) where addition of N in the media resulted in higher biomass density eventually resulting in higher CP utilization.

There was evidence of a slight reduction of CP concentration in MSM without P (+N – P + C) suggesting a capacity of the bacterial strains to degrade CP as a source of P but this was not statistically significant, despite the fact that the same enzyme phosphotriesterase catalyzes the hydrolysis of P–O–C linkage of OP compounds (as evident from TCP accumulation). In one study, Shelton (1988) isolated a bacterial consortium that could use diethylthiophosphoric acid as a C-source only and was unable to degrade it as a P- or sulfur (S)-source. A possible explanation is that most often a particular compound is used to supply only a single element and the way in which the metabolism (degradation) is regulated depends strongly on the organism and OP compound studied (Kertesz et al., 1994). The results suggested that the bacterial isolates could utilize CP and its primary metabolite (TCP) as both C- and N-sources but not as a source of P. This is the first time that species of *Xanthomonas, Pseudomonas* and *Rhizobium* has been shown to be able to utilize CP as a source of both C and N.

Previous research on CP degradation reported that the removal of CP resulted in the formation of metabolites like CP-oxon, 3, 5, 6-trichloro-2-methoxypyridine, and 2-chloro-6-hydroxypyridine (Singh et al., 2006; Yu et al., 2006). Although *Enterobacter* strain B-14 degraded 40% of 25 mg/L CP within 48 h (Singh et al., 2004), *Stenotrophomonas* sp. YC-1 degraded 100% of 100 mg/L within 24 h (Yang et al., 2006) and *Synechocystis* sp. PUPCCC 64 degraded 93.8% of 5 mg/L CP within 5 days (Singh et al., 2011), these strains failed to utilize TCP for growth and energy. However, previous literature has reported certain bacterial strains capable of also degrading TCP produced as a primary CP degradation product. *A. faecalis* DSP3 degraded 100% and 93.5% of 100 mg/L CP and TCP within 12 days (Yang et al., 2005), *Paracoccus* sp. TRP degraded 50 mg/L CP and TCP in 4 days (Xu et al., 2008), *B. pumilus* C2A1 degraded 89% of 1000 mg/L CP within 15 days and 90% of 300 mg/L TCP within 8 days (Anwar et al., 2009), *Mesorhizobium* sp. HN3 degraded 100% of 50 mg/L CP and TCP (Jabeen et al., 2015). In the present study, only transient accumulation of TCP was observed by *Xanthomonas* sp. 4R3-M1 and *Pseudomonas* sp. indicating degradation of both CP (20 mg/L) and TCP were achieved by the same isolate within 6 days of incubation. This was further confirmed by the degradation and growth on TCP, when added externally.

In contrast, CP degradation by *Rhizobium* sp. 4H1-M1 did not show formation of TCP in any of the media types. It suggests that this bacterial strain might be utilizing some alternative mechanism for CP degradation for intracellular pathways, with the help of endo-enzymes. Similar results were obtained in a microcosm study where no TCP formation occurred during CP degradation by *Bacillus* species (Lakshmi et al., 2008). The ability of *Rhizobium* strains to produce exo- and endo-cellular phosphodiesterase and phosphotriesterase, and to participate in the hydrolytic detoxification of OP pesticide, was first studied in 1994 (Abd-Alla, 1994). Considering the magnitude of toxicity of TCP, an organism that uses an intracellular pathway for degrading TCP would be well-suited for bioremediation of contaminated sites. It is worth mentioning here that *Rhizobium* bacteria are known for their ability to colonize roots of legumes and assist in the growth of host plant by fixing atmospheric nitrogen. Thus, there exists a potential for application of CP and TCP-degrading *Rhizobium* strains to clean-up contaminated system and maintain soil health.

Degradation of TCP, when added externally, occurred with *Xanthomonas* sp. 4R3-M1 and *Pseudomonas* sp. 4H1-M3 when inoculated in various liquid media composition. The finding revealed that both *Xanthomonas* sp. 4R3-M1 and *Pseudomonas* sp. 4H1-M3 utilized TCP as a source of C while they were grown solely on TCP or supplementation with an alternate C-source. A similar result was observed for an isolate of *Pseudomonas* sp. capable of TCP degradation (Feng et al., 1997). The disappearance of TCP occurred in all MSM media except for MSM without phosphorus (+N – P + C) with increases in OD_600_ indicating that TCP was utilized as sources of N and C for growth by *Xanthomonas* sp. 4R3-M1 and *Pseudomonas* sp. 4H1-M3 (discussed above), even when added externally to the media.

The strains showed reduced degradation of TCP in MSM (+N – P + C) accompanied by a poor microbial growth. These results suggested that although the strains were utilizing TCP for C and N, the limitation of phosphorus in the media caused the biomass to decrease (Todar, 2013). *Rhizobium* sp. 4H1-M1 appeared to be a potential degrader of CP (and its primary metabolite) but failed to utilize TCP for growth and energy when TCP was added externally into the liquid media. Since very little literature is available about TCP metabolism it is difficult to conclude anything. However, it can be argued that in this particular strain, CP enters the cell and is intracellularly degraded as a C- and N-source along with its primary metabolite, TCP. It is possible that this strain does not possess mechanisms or enzyme-linked receptors to transport externally added TCP for intracellular degradation and thus no degradation of externally added TCP was observed. However, further work is required to confirm this. Similar to CP degradation, this is the first study to report bacterial strains from genera *Xanthomonas* and *Pseudomonas* to utilize TCP as source of both C and N.

## Conclusion

In the present work, novel CP and TCP-degrading bacterial strains were isolated from sugarcane farm soils that showed enhanced degradation of CP in a laboratory-based study. All strains isolated were able to grow in the presence of CP and/or TCP and were able to degrade CP and/or TCP as C, N, and P sources. This is the first report to show the simultaneous use of CP and/or TCP as a C and N source by any bacterial strain. The use of such efficient indigenous bacterial strains promises to be effective in practical application of bioremediation of both CP and TCP since the microbes have already adapted to the localized habitat conditions. The isolated strains can also be added to other soils as microbial inoculants (bio-augmentation) for their potential to degrade pesticides to improve soil quality in order to create a more sustainable agriculture and environment. These strains can also provide new OP-degrading genes, enzymes and pathways to be harnessed for a range of biotechnological and other applications, such as, enzyme-based remediation and treatment of OP poisoning in human beings. This study provides novel insights and promising organisms for bioremediation, however, further studies are needed before successful implementation of bioremediation on the efficacy of such organisms and their survival in the contaminated environment.

## Author Contributions

BS conceived the idea, SR did all experimental works and analyzed data in consultation with all co-authors. SR wrote the first draft with inputs from all other authors.

## Conflict of Interest Statement

The authors declare that the research was conducted in the absence of any commercial or financial relationships that could be construed as a potential conflict of interest.

## Footnotes

1 blast.ncbi.nlm.nih.gov/

2 http://www.ncbi.nlm.nih.gov
