# Supplementary material for: Isolation and Molecular Characterization of Novel Chlorpyrifos and 3,5,6-trichloro-2-pyridinol-degrading Bacteria from Sugarcane Farm Soils
Source: Front Microbiol. 2017 Apr 4;8:518. doi: 10.3389/fmicb.2017.00518 (PMC5378769; doi:10.3389/fmicb.2017.00518)
Supplement: Supplementary file 1 [file Table_1.docx]

Supplementary Table 1: Pesticide history of the five sugarcane farms (Queensland, Australia) used in this study.

| **Site** | **Region** | **History of use of sugarcane grub control products***  **(past pesticide treatment)** |
| --- | --- | --- |
| 1 | Burdekin I  (latitude -19.59683^o^,  longitude 147.38606^o^) | Clay loam soil that had rapid degradation/greyback grub failures following use of suSCon Blue^a^ in 1990’s. suSCon Maxi^b^ (15 kg/ha) subsequently used 3 times per year since permitted in 2002 |
| 2 | Burdekin II  (latitude -19.86424^o^,  longitude 147.24409^o^) | River loam soil that had rapid degradation/greyback grub failures following use of suSCon Plus^c^ in 1990’s. suSCon Maxi (14 kg/ha) subsequently used 2 times per year since permitted in 2002 |
| 3 | Mackay  (latitude -21.12813^o^,  longitude 149.06880^o^) | Red clay soil where suSCon Blue is still successfully used for French’s sugarcane grub control and has been used at least 2-3 times per year in the last 12 years. |
| 4 | Burdekin III  (latitude -19.86424^o^,  longitude 147.24409^o^) | River loam where there has been use of suSCon Blue since 1990’s. suSCon Maxi (15 kg/ha) subsequently used 2 times per year since permitted. |
| 5 | Tully  (latitude -19.59683^o^,  longitude 145.94784^o^) | Sandy loam soil with history of suSCon Blue treatment in 1998 and 2001. suSCon Maxi (15 kg/ha) subsequently used 3 times per year since permitted. |

(*) data available till the year 2012

suSCon is the commercial product name of Crop Care (Australia)

^a^active ingredient chlorpyrifos (140 g/kg)

^b^ingredient imidacloprid (50 g/kg).

^c^active ingredient chlorpyrifos (75 g/kg)
